# Understanding mechanisms of depression prevention: study protocol of a randomized cross-over trial to investigate mechanisms of mindfulness and positive fantasizing as intervention techniques for reducing perseverative cognition in remitted depressed individuals

**DOI:** 10.1186/s12888-024-05592-8

**Published:** 2024-02-19

**Authors:** Marlijn E. Besten, Marieke van Vugt, Harriëtte Riese, Claudi L. H. Bockting, Brian D. Ostafin, André Aleman, Marie-José van Tol

**Affiliations:** 1 Department of Biomedical Sciences of Cells and Systems Cognitive Neuroscience Center, University Medical Center Groningen, Groningen, The Netherlands; 2 Department of Clinical Neuropsychology, Faculty of Behavioural and Social Sciences, University of Groningen, Groningen, The Netherlands; 3 Computer Science and Artificial Intelligence, Bernoulli Institute of Mathematics, University of Groningen, Groningen, The Netherlands; 4 Department of Psychiatry, Interdisciplinary Center Psychopathology and Emotion Regulation, University Medical Center Groningen, Groningen, The Netherlands; 5 Department of Psychiatry, Academic Medical Center, Amsterdam, The Netherlands; 6 Centre for Urban Mental Health, University of Amsterdam, Amsterdam, The Netherlands; 7 Department of Psychology, University of Groningen, Groningen, The Netherlands

**Keywords:** Depression, Relapse, Rumination, Mindfulness, Positive fantasizing, Preventive cognitive therapy, Perseverative cognition

## Abstract

**Background:**

Major Depressive Disorder (MDD) is one of the most prevalent psychiatric disorders, and involves high relapse rates in which persistent negative thinking and rumination (i.e., perseverative cognition [PC]) play an important role. Positive fantasizing and mindfulness are common evidence-based psychological interventions that have been shown to effectively reduce PC and subsequent depressive relapse. How the interventions cause changes in PC over time, is unknown, but likely differ between the two. Whereas fantasizing may change the valence of thought content, mindfulness may operate through disengaging from automatic thought patterns. Comparing mechanisms of both interventions in a clinical sample and a non-clinical sample can give insight into the effectivity of interventions for different individuals. The current study aims to 1) test whether momentary psychological and psychophysiological indices of PC are differentially affected by positive fantasizing versus mindfulness-based interventions, 2) test whether the mechanisms of change by which fantasizing and mindfulness affect PC differ between remitted MDD versus never-depressed (ND) individuals, and 3) explore potential moderators of the main effects of the two interventions (i.e., what works for whom).

**Methods:**

In this cross-over trial of fantasizing versus mindfulness interventions, we will include 50 remitted MDD and 50 ND individuals. Before the start of the measurements, participants complete several individual characteristics. Daily-life diary measures of thoughts and feelings (using an experience sampling method), behavioural measures of spontaneous thoughts (using the Sustained Attention to Response Task), actigraphy, physiological measures (impedance cardiography, electrocardiography, and electroencephalogram), and measures of depressive mood (self-report questionnaires) are performed during the week before (pre-) the interventions and the week during (peri-) the interventions. After a wash-out of at least one month, pre- and peri-intervention measures for the second intervention are repeated.

**Discussion:**

This is the first study integrating self-reports, behavioural-, and physiological measures capturing dynamics at multiple time scales to examine the differential mechanisms of change in PC by psychological interventions in individuals remitted from multiple MDD episodes and ND individuals. Unravelling how therapeutic techniques affect PC in remitted individuals might generate insights that allows development of personalised targeted relapse prevention interventions.

**Trial registration:**

ClinicalTrials.gov: NCT06145984, November 16, 2023.

**Supplementary Information:**

The online version contains supplementary material available at 10.1186/s12888-024-05592-8.

## Background

Major Depressive Disorder (MDD) is one of the most prevalent psychiatric disorders affecting about 20% of the world population at some point in their life [1, 2]. The risk of a relapse after suffering from a depressive episode is high (40–60% after recovering from a first episode [3–5]. Therefore, lowering relapse vulnerability is an important therapeutic target. Understanding how specific psychological interventions affect core vulnerability factors is a potentially powerful way to improve individual patient’s relapse prevention.

Ruminative thinking is one of the core factors creating high vulnerability for relapse as rumination and related worrying have been identified as key processes in developing and maintaining MDD [6, 7]. MDD patients report more negative, past-related, and self-related spontaneous thinking compared to healthy individuals [8]. MDD patients also frequently engage in ruminative thinking, characterized by repetitive, negative, and uncontrollable thoughts that are difficult to disengage from [9]. This type of thinking can also be put under the umbrella term ‘perseverative cognition’ (PC) [6, 10]. PC often remains after remission from a depressive episode and is a common residual symptom (e.g., [7, 11]). Because of its relation with depressive relapse [7, 12], targeting PC in the remitted stage of MDD could be a powerful way of preventing the recurrence of depression.

Two psychological interventions, namely *positive fantasizing* and *mindfulness*, are core therapeutic components of multiple sessions protocolized treatments. Preventive Cognitive Therapy (PCT) delivered during the remitted phase has proven effective in lowering relapse risk [13–19]. Positive fantasizing is a core component of PCT that is aimed at challenging dysfunctional attitudes and schemas by using positive phantasy with help of imagery, enhancing positive affect and positive cognitions. Dysfunctional attitudes are attitudes or beliefs that lead individuals to engage in negative, self-referential thinking (i.e., PC) [20, 21]. By changing dysfunctional attitudes with positive fantasizing, we expect PC to change as well. Other components of PCT are enhancing positive autobiographical memories, and designing a personal prevention plan [22]. Mindfulness—which in the context of Mindfulness Based Cognitive Therapy has been shown to be effective at lowering depressive vulnerability [23–26]— aims to change dysfunctional attitudes and schemas by increasing awareness of the present moment and training an individual to disengage from automatic thought patterns (such as PC) and to develop a non-judgmental attitude towards all mental content. Both intervention techniques have proven to exert effects already in a single-session exercise. Specifically, single-session mindfulness exercises have been shown potent at reducing rumination and depressive symptoms [27, 28] and a ten-minute fantasizing exercise increased positive affect and decreased negative affect [29] in non-depressed individuals. Moreover, when contrasted with thinking after a 10-min stress-induction intervention, 10-min positive fantasizing resulted in thoughts that were less negative and more positive and less past- and more future-oriented [29]. It is however not sure whether these techniques lead to longer-lasting effects in a longer intervention.

To unravel how mindfulness and positive fantasizing affect PC in individuals vulnerable for depression, detailed measurements of the content and dynamics of PC is essential. Behaviourally, PC can be assessed by retrospective self-report covering self-assessment of cognitive behaviour on multiple timescales, using scales of rumination covering weeks (e.g., [30, 31]) or by individual momentary items (e.g., “Right now, how difficult was it to disengage from the thought?”) [32] covering minutes, respectively. Moreover, earlier studies lack task-based cognitive measures to assess PC, which could be helpful for understanding of how PC is changed on a behavioural level [32]. Moreover, PC has been found to be associated with psychological measures that may clarify the role of the central nervous system during PC. Specifically, increased heart rate (HR), reduced heart rate variability (HRV) [10, 33, 34], and aberrant brain activity [35–39] during PC was found. Therefore, HR, HRV, and electroencephalogram (EEG) may constitute (neuro)physiological measures of PC at millisecond-to-second timescales.

The current study will combine momentary-, behavioural-, cognitive-, and physiological measures at different timescales, which could give insight into the manifestations and dynamics of PC and how it can be modified by interventions. The effects of positive fantasizing and mindfulness will be contrasted by means of affect, cognition and physiology, all sampled at high temporal density, to see whether those can clarify different mechanisms for reducing PC. Whereas positive fantasizing is potent in reducing PC by using positive phantasy to enhance positive affect and positive cognitions, mindfulness focusses on increasing awareness of the present moment and is therefore potent in reducing PC. Additionally, we will characterize sleep patterns using actigraphy and self-report. Previously, PC has been found associated with poor sleep quality and sleep onset latency, which in itself can increase PC [40]. By including individual characteristic variables (such as personality, depressive history, and childhood trauma) as potential moderators of the main effect of intervention on PC, we can gain insight in what intervention works best for whom. This could be a step towards better personalized treatment [41]. The overall aim of the current study is to study the mechanisms by which mindfulness and positive fantasizing may affect the content and dynamics of PC and thereby contribute to lower depressive relapse vulnerability. The study has three objectives.

The first objective is to study the differential mechanisms by which mindfulness and positive fantasizing affect PC in individuals vulnerable for depressive relapse. To this end, we will randomize 50 remitted MDD (rMDD) individuals in a cross-over study to both mindfulness and fantasizing, with the order of interventions randomized over participants and the same duration of the interventions. We hypothesize that both techniques change PC but that they exert these effects differently. Specifically, we expect positive fantasizing to obtain its effects by increasing positive affect and changing the content of thoughts and expect mindfulness to obtain its effects by increasing focus on the present moment.

The second objective is to examine whether effects of mindfulness and fantasizing on PC in rMDD individuals differ from ND individuals. To this end, 50 ND participants will be matched to the rMDD participants and similarly randomized to undergo both mindfulness and fantasizing interventions. Comparing the effects of interventions in both rMDD and ND individuals allows us to study whether therapeutic techniques, developed and often studied in a clinical sample, work similar or different in ND individuals. Since most studies focus on the effects of mindfulness and positive fantasizing in a clinical sample [16, 19, 27, 28, 42], we hypothesize these techniques to be effective in reducing PC in rMDD individuals but expect that the interventions may show different effects on PC in ND individuals.

The third objective is to examine in an exploratory manner whether individual characteristics affect the effectiveness of the two interventions in reducing PC across all participants (i.e., what works for whom). We know from the previous literature that there is substantial variability in treatment response between MDD individuals [43,44﻿]. This variability has been suggested to be related to individual characteristics such as depressive symptoms and history, comorbid disorders/symptoms, personality traits, and cognitive dysfunction. For example, people with anhedonia may have a harder time to engage in positive fantasizing, and people with lower levels of executive functioning (mental flexibility) may find mindfulness practice difficult. Whether these independent predictors of depressive vulnerability affect intervention effects is interesting for personalized treatment purposes. This objective is exploratory, and hence we do not have specific hypotheses about the direction of these effects.

## Methods

### Design

**Fig. 1:**
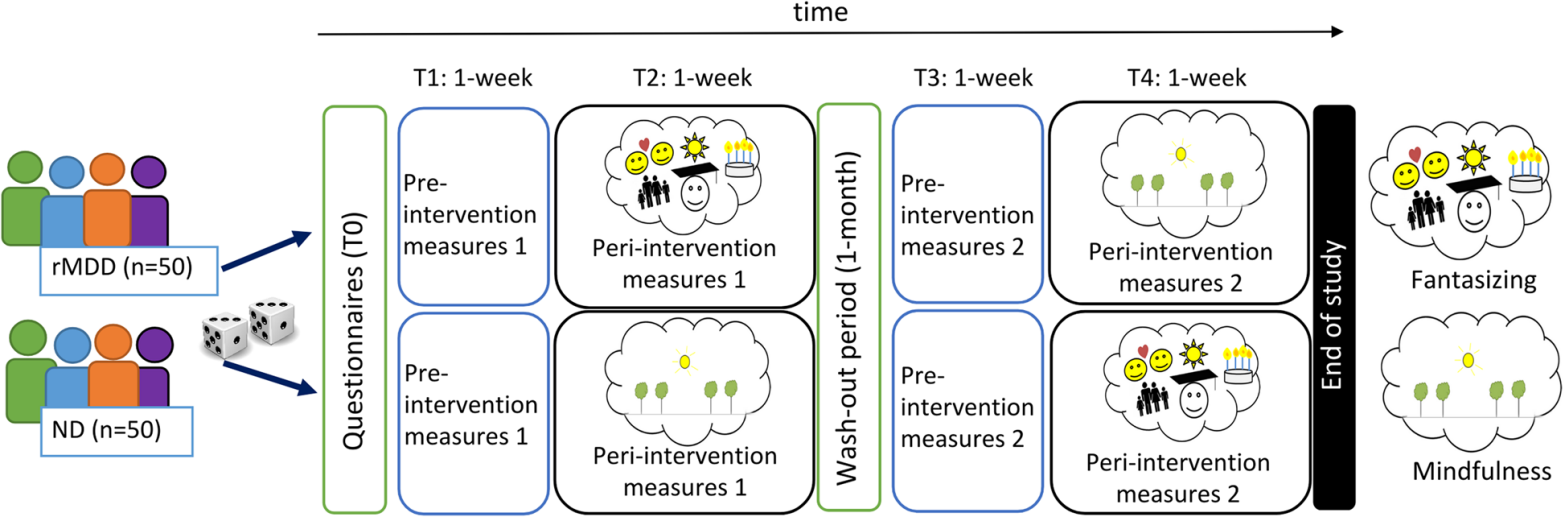
Flowchart of the MINDCOG study

To examine the differential effects of mindfulness and positive fantasizing in rMDD and ND participants, we designed an open-label intervention cross-over study in which participants received both a mindfulness- and fantasizing intervention, in randomized order. The study is conducted at the University Medical Center Groningen, the Netherlands. The study includes five phases after an initial screening that is used to determine study eligibility. Eligible participants undergo 1) an assessment of baseline characteristics using self-report questionnaires (T0); 2) pre-intervention measurement 1 including one week of baseline momentary measures using Experience Sampling Methods (ESM) ten times per day, cognitive task performance two times per day, 24-h measurements of impedance cardiography (ICG)/electrocardiogram (ECG), one-week actigraphy measurements and an EEG lab measurement and questionnaires in the lab (T1); 3) peri-intervention measurement 1 where participants practice daily with an intervention and undergo the same measurements as in pre-intervention measurement 1 (T2); 3) pre-intervention measurement 2, which takes place after a wash-out period of at least one month consisting of one-week momentary measures, two-times daily cognitive task performance, actigraphy, 24-h of ICG/ECG, and EEG measurements and questionnaires in the lab (T3); 4) peri-intervention measurement 2, including practicing with the not-yet-performed intervention and the same measurements as in pre-intervention measurement 2 (T4). An overview of the study design can be found in Fig. 1.

### In- and exclusion criteria

#### Inclusion criteria

In order to be eligible to participate in this study, all participants must meet all the following criteria:

- Participants should be between 18 and 60 years old. They should not exceed 60 years of age in order to minimize aging-related decline in information processing [45];
- Participants should display normal intelligence (IQ > 85, as assessed with the Dutch Adult Reading Test (DART) [46] and/or having finished an education on at least vocational level) in order to assure sufficient task comprehension.

Participants in the rMDD group should meet the following criteria to make sure that they are at high risk of depressive relapse and currently show no clinically relevant severity of depressive symptoms:

- Remitted participants should have experienced at least two depressive episodes, according to criteria defined by the Diagnostic Statistical Manual, version 5 (DSM-5), experienced in past ten years;
- Remitted participants should score 21 or lower on the Inventory of Depressive Symptomatology - Self-Report (IDS-SR30) [47], indicative of the absence of clinically relevant depressive symptoms.

#### Exclusion criteria

Furthermore, individuals who meet any of the following criteria are excluded from participation in this study:

- Fulfilling criteria for any current DSM-5 diagnosis as objectified with the Structured Clinical Interview for DSM-5 (SCID-5) [48];
- Daily use of anti-depressive medication, benzodiazepines, methylphenidate, beta blockers or other medication potentially influencing ICG/ECG currently or in the last four weeks;
- Recent engagement (defined as in their last episode, or as one year prior to inclusion in case the last episode was more than a year before inclusion) in PCT including the positive fantasizing technique and/or have recent experiences (defined as daily practice in the past two years for at least two weeks) with mindfulness, meditation, or mindful yoga. This criterion prevents underestimation of true effects of the interventions and maximizes treatment effects;
- Participation in another clinical intervention study at the moment of inclusion in the study to prevent overlapping intervention effects.

Individuals for the ND control group who additionally meet any of the following criteria are excluded from participation in this study:

- Presence of symptoms of depression according to the IDS-SR30 (score > 13), to make sure they are not currently experiencing clinically relevant depressive symptoms;
- Any life-time psychopathology of any disorder as objectified with the SCID-5.

### Sample size

To our knowledge, we are the first to combine ESM, ICG/ECG measures, and laboratory EEG measures to study the effects of different interventions on PC. It is difficult to do power calculations for our study since there is no software available that can make predictions for our chosen statistical methods (linear mixed effect models & generalized additive models; see Statistical Analyses). We therefore calculated the sample sizes using G*Power (version 3.1.9.4) for t-tests based on questionnaire measures.

In order to study the differential effects between mindfulness and positive fantasizing on PC in individuals vulnerable for depressive relapse, we will include 50 rMDD participants. In a meta-analytic review, Perestelo-Perez et al. [49] compared the effects of Mindfulness-Based Cognitive Therapy with usual care on rumination in patients with a current depressive disorder or a depressive disorder in the past. They observed a Hedges’ *g* of -0.59 for the effect of Mindfulness-Based Cognitive Therapy compared to usual care in reducing ruminative thinking. Using G*Power 3.1.9.4, sample sizes based on these outcomes were calculated. We expect PC to reduce after mindfulness and positive fantasizing. Therefore the sample size for one-tailed t-tests was calculated. To conduct one-tailed t-tests with a statistical power of 80%, the sample size should be *n* = 36 participants. A sample size of *n* = 50 rMDD participants should therefore give sufficient power to draw reliable conclusions about treatment effects, especially since questionnaires are a less sensitive measure than most of the other measurements used in the current study (i.e., ESM, ICG/ECG and laboratory EEG). This relatively large sample size also helps us to buffer against inevitable data losses related to e.g., participants not completing all questionnaires or technical malfunctions of the physiological measurements.

To conduct reliable between-group comparisons on how the effects of mindfulness and fantasizing on PC differ between rMDD and ND individuals, we will include 50 ND participants that are matched to the 50 rMDD participants on age, sex, and education level. In a study of Lois and Wessa [50], they compared self-reported rumination scores between rMDD individuals and matched healthy controls at a baseline level. They observed a t-value of *t*(48) = 2.4 (effect size Cohen’s *d* = 0.686) comparing rumination scores between rMDD participants and controls. Based on these outcomes, sample sizes were calculated using G*Power 3.1.9.4. No specific direction of effects was hypothesized for the effects of mindfulness and fantasizing on PC in rMDD versus ND individuals. Therefore, the sample size for two-tailed t-tests was calculated. To conduct two-tailed t-tests with a statistical power of 80%, sample sizes should be *n* = 28 participants per group. This suggests a sample size of *n* = 50 participants per group should give sufficient power to draw reliable conclusions about between-group differences in changes in PC.

### Recruitment

Participants are recruited via advertisements campaigns on (social) media, posters, and flyers in public spaces and patient organizations. After inclusion of a rMDD participant, a ND control participant is matched based on their age, sex, and education level.

### COVID-19 related changes to the study design

At the time of data collection (March 2020), the COVID-19 pandemic started which led to a delay in the data collection. We therefore had to change the original protocol in order to collect data of a sufficiently large sample within the funding period. We decided to split the study into two phases: phase 1) including the original measurements as written in this protocol paper but with 25 rMDD and 25 ND control participants and phase 2) including the questionnaires, cognitive task and ESM measurements as described in this protocol paper but without the physiological measurements in the form of an online study with *n* = 25 participants per group. This way, we will have a sample of *n* = 25 participants per group for the EEG, ECG/ICG, and actigraphy data and a sample of *n* = 50 participants per group for the ESM, questionnaires, and cognitive task data. Data collection to reach the desired sample size for these two phases is still ongoing and expected to finish in June 2024.

The power analyses of the original study were calculated based on questionnaire data. The sample size for the questionnaire, cognitive task and ESM data will therefore not change. For the physiological measurements (laboratory EEG, ambulatory ECG/ICG, and actigraphy; part 1) we performed a new power analysis. Our power analysis is based on a study of Jin et al. [37], which has a similar EEG task setup as in the current study. They examined differences between event-related potentials related to on-task behaviour versus mind-wandering behaviour which is similar to our intended analysis of comparing ruminative vs. non-ruminative trials [51]. They found a difference in the P3 component between being on-task and when mind-wandering [*t* = 3.97, *p* < 0.001, *d* = 0.59]. Using G*Power 3.1.9.7, a target sample size based on this result was calculated. To conduct a one sample t-test with a statistical power of 80%, a sample size of *n* = 20 participants is needed to draw reliable conclusions about treatment effects on PC measured with EEG.

For the physiology, we rely on a study of Ottaviani et al. [52] in which the differences between HR in individuals with MDD and healthy controls were examined. They found a significant difference in HR between MDD- and healthy control-participants with an effect size of *d* = 0.96. This effect size was used to calculate the sample size using G*Power 3.1.9.7. To conduct two-tailed t-tests with a statistical power of 80%, a sample size of 19 participants per group is sufficient to draw reliable conclusions.

Together, this suggests that a sample size of *n* = 25 participants per group for (neuro)physiological measurements is sufficient to examine the effects of interventions and differences between the rMDD and ND groups.

### Interventions

#### Mindfulness

Mindfulness-Based Cognitive Therapy is an eight-week intervention, consisting of eight training sessions of around two hours and daily practice at home of 30–40 min [24]. The intervention aims at increasing mindfulness and/or decreasing negative repetitive thoughts, and has been shown to reduce depressive relapse rates, depressive symptoms, and rumination [23, 25, 26]. In this study, we isolate two important aspects of Mindfulness-Based Cognitive Therapy, namely the professional training and short daily exercises.

In the current study, participants receive one professional training session (2 h) in the intervention in groups of 2–8 people to become familiar with the techniques, learn the basics needed for the daily exercises, and get questions answered. The training is given by a mindfulness professional and consists of psycho-education, instructions for mindfulness, and guided practice. It focuses mainly on attending to stimuli such as breathing, external sounds or bodily sensations, and becoming aware of where one’s attention is. For practical reasons, we work with two mindfulness professionals that alternate the training sessions.

After the professional training, participants receive instructions about the audio application with which they continue performing short exercises using the learned technique every day guided by an application on their smartphone customized for this research. Specifically, participants perform one short exercise (10 min) per day for in total six days. The exercises that are being performed are:

- day 1: attention to breathing part 1;
- day 2: attention to breathing part 2;
- day 3: attention to sounds;
- day 4: attention to bodily sensations;
- day 5: attention to thoughts;
- day 6: attention to emotions and feelings.

#### Positive fantasizing

The positive fantasizing technique is part of the PCT intervention [22]. PCT has been shown to be effective in preventing for depressive relapse and reducing depressive symptoms [13, 15, 16, 18, 19, 42]. In our previous study [29], we isolated a short fantasizing exercise of ten minutes from the full protocol, and found that a single session was effective at reducing negative affect when measured directly after the single-session intervention.

In the current study, we isolated the positive fantasizing technique from the PCT. In a two-hour training session by a professional trainer, participants get familiar with the positive fantasizing technique. Participants receive psycho-education about the role of dysfunctional “beliefs and schema”. The positive fantasizing technique as derived from PCT starts with identifying a dysfunctional “beliefs and schema” and subsequently fantasize about a positive “fantasy belief”. This fantasy belief can be the extreme opposite of the dysfunctional belief, but also can be a different extreme positive belief. For example, instead of “I am worthless”, participants are invited to consider the belief: “I am wonderful”. Participants are guided by the professional in choosing their limiting belief and the opposite, extremely positive belief which they investigate using the fantasizing technique. Then, participants are guided by the professional using imagery and experiencing the thoughts and feelings that would be elicited when using this, as if in an ideal world. It is made very clear that the situation they are visualizing does not have to be realistic. After using imagery techniques, participants are asked, with help of the professional, to reflect on their experience during the fantasizing exercise and to think of how they could implement this fantasy belief more in their daily life, by adapting their fantasy belief into a more practical belief, in line with how the technique is used in PCT. When participants have learned the basics of the fantasizing technique, the participants write down, together with the professional trainer, what they will be fantasizing about at home. They use the same belief every day but they can choose to apply the rule to different life situations.

Participants are asked to perform one exercise per day using the fantasizing technique (10 min), via a mobile application on their mobile phone for six days in total. Participants are guided by an audio voice leading the fantasizing exercise (e.g., asking questions such as: “Describe and imagine what it would be like if you were to live according to your fantasy belief”).

The time, instructions, and exercises are comparable for both interventions. The date and time of the exercise sessions are logged on a phone app which allows us to determine the amount of actual practice a participant engages in.

### Randomization

All participants receive both interventions, which intervention they receive first is pseudo-randomly counterbalanced across participants. Pseudo-randomization here means that individuals are randomized to either fantasizing or mindfulness as first intervention by order of inclusion. Specifically, every three weeks a new measurement session starts. Mindfulness and fantasizing alternate in three-week blocks. New sign-ups in this three-week block are allocated to mindfulness or fantasizing, in alternating order.

### Procedure

**Fig. 2:**
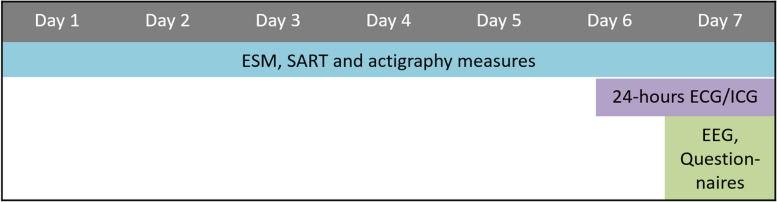
Overview of a pre-intervention measurement session (T1, T3)

**Fig. 3:**
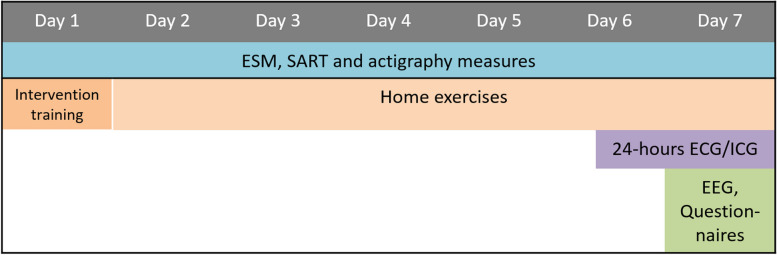
Overview of a peri-intervention measurement session (T2, T3)

After individuals have shown interest in participation in the study and are fully informed, they are asked to provide written informed consent. After providing informed consent, participants are screened for eligibility based on outcomes of the SCID-5, DART, IDS-SR30 and some sociodemographic background questions (see In- and exclusion criteria). When found eligible, they are asked to participate in the actual experiment that consists of five phases: a baseline individual characteristics measurement (T0) and four pre- and peri-intervention measurements periods (T1-T4; See Table 1, Figs. 2 and 3).

**Table 1:** Overview of the MINDCOG assessments and their timing, where T0 refers to baseline characteristics before the start of the measurement period and T1-T4 the pre- and peri-intervention measurement sessions. More detailed information on the timing of the measurements assessed in T1-T4 can be found in Figs. 2 and 3

	**Screening**	**T0**	**T1**	**T2**	**T3**	**T4**
SCID-5 Interview	x					
IDS-SR30	x		x	x	x	x
DART	x					
AES		x				
BVAQ		x				
LEIDS-RR		x				
CTQ-SF		x				
DAS-A		x				
NEO-FFI		x				
ESM			x	x	x	x
Actigraphy			x	x	x	x
EEG			x	x	x	x
ECG/ICG			x	x	x	x
PTQ			x	x	x	x
RPA			x	x	x	x
ERQ			x	x	x	x
FFMQ			x	x	x	x
LARSS			x	x	x	x
PANAS			x	x	x	x
Expectation interview interventions			x			
Evaluation interview intervention				x		x

Abbreviations used in the table. *(SCID-5)* Structured Clinical Interview for DSM-5, *(IDS-SR30)* Inventory of Depressive Symptomatology - Self-Report, *(DART)* Dutch Adult Reading Test, *(AES)* Apathy Evaluation Scale, *(BVAQ)* Bermond-Vorst Alexithymia Questionnaire, *(LEIDS-RR)* Leiden Index of Depression Sensitivity-2nd revision, *(CTQ-SF)* Childhood Trauma Questionnaire-Short Form, *(DAS-A)* Dysfunctional Attitude Scale-form A, *(NEO-FFI)* NEO Five-Factor Inventory, *(ESM)* experience sampling method, *(EEG)* electroencephalogram, *(ECG)* electrocardiogram, *(ICG)* impedance cardiogram, *(PTQ)* Perseverative Thinking Questionnaire, *(RPA)* Responses on Positive Affect Scale, *(ERQ)* Emotion Regulation Questionnaire, *(FFMQ)* Five Facet Mindfulness Questionnaire, *(LARSS)* Leuven Adaptation of the Rumination on Sadness Scale, *(PANAS)* Positive and Negative Affect Schedule

#### Baseline individual characteristics (T0)

As baseline measurement (T0) self-report questionnaires are administered to measure individual characteristics that could serve as individual markers to predict treatment efficacy. Based on factors associated with the longitudinal course of depression, these include personality [44, 53], attitudes [21], depression sensitivity [54], childhood trauma [44], alexithymia [55], and apathy [56]. We administer the NEO Five-Factor Inventory (NEO-FFI) [57], Dysfunctional Attitude Scale-form A (DAS-A) [21], Leiden Index of Depression Sensitivity-2nd revision (LEIDS-RR) [58], Childhood Trauma Questionnaire-Short Form (CTQ-SF) [59], Bermond-Vorst Alexithymia Questionnaire (BVAQ) [60], and Apathy Evaluation Scale (AES) [56] and some questions about their expectations about the interventions. This takes approximately 65 min. Questionnaire data is collected and stored using REDCap.

#### Pre- and peri-intervention measurements procedure (T1-T4)

A few days before the start of the pre- and peri-intervention measurements, participants receive instructions about how to perform the measurements. The pre-and peri-intervention measurements include momentary ESM, the Sustained Attention to Response Task (SART), actigraphy, ICG/ECG measures, laboratory EEG measures, and questionnaires. The measurements are the same for each pre-and peri-intervention session.

##### ESM

Participants complete ESM questionnaires about sleep, feelings, thoughts, environment, and events using ESM via a mobile phone. In total, six ESM items about sleep are administered once per day and 24 ESM items are administered ten times per day for seven days in total. Filling out these questionnaires takes approximately five minutes. An overview of the ESM items can be found in Supplementary Materials 1. Data is collected and stored using RoQua, a highly protected system to manage and collect ESM data. Information on the dynamics and content of self-reported PC are extracted from the ESM data using the following variables: rumination, occurrence of off-task thinking, valence of thoughts, temporal orientation of thoughts, self-relatedness of thoughts, and stickiness of thoughts (i.e., how difficult it is to disengage from the thought).

##### SART

Participants perform a short version of the SART adapted from McVay and Kane [61], developed for use via a mobile application. The SART is a cognitive Go/No-Go task combining self-report measures of PC with task performance. The SART is performed twice-a-day for seven days and takes approximately five minutes to perform. Data is collected and stored using a protected server of the University of Groningen, the Netherlands. The task includes four blocks with four thought probe questions (asking about the content, valence, temporal orientation, and stickiness of the current thought) per block. Both self-reported PC (extracted from probe questions on the content, valence, temporal orientation and stickiness of the current thought) and task-performance (i.e., response times and accuracy) are extracted from the SART and used for further analyses.

##### Actigraphy

Actigraphy is measured with an epoch every 60 s for seven days using a MotionWatch 8 (CamNtech; www.camntech.com/motionwatch-8/). Data is stored on a shielded drive and pre-processed using the CamNtech MotionWare software. Sleep variables including total sleep time, sleep quality, and sleep onset latency are extracted from the data using the MotionWare software.

##### ECG/ICG

Participants are asked to wear an ambulatory cardiac monitoring system (VU-AMS; www.vu-ams.nl) [62, 63] for 24-h that allows us to measure ICG and ECG. In the peri-intervention measurement period, the VU-AMS device is worn on the last 24-h of the measurement period. Participants can apply the devices themselves using instructions or with remote help of the research team. The VU-AMS system contains seven electrodes, three ECG electrodes and four ICG electrodes. Data is sampled at a frequency of 1000 Hz and stored on a shielded drive. After data collection, the data is pre-processed using the VU-AMS software and matched with participants’ self-reported PC derived from the ESM data. An interval of 5-min before the time of the ESM beep in which participants reported PC is marked in the ECG to study the psychological correlates of self-reported PC.

##### EEG

On the last day of the measurement week, participants visit the lab where EEG and ECG measurements are performed during resting-state, an implicit emotion regulation task, and SART. EEG and ECG data in the lab is recorded using a BioSemi Active Two system (www.biosemi.com) with 32 electrodes positioned at the 10–20 system and eight external electrodes [64]. Data recording is done using the Actiview software, sampled at a 512 Hz frequency and stored on a shielded drive. Data is pre-processed using EEGLAB [65]. The cognitive tasks are used to study the physiological correlates during task performance, comparing PC thoughts with non-PC thoughts using the SART and studying the physiological correlates of different emotion processing strategies using the emotion regulation task. Specifically, we extract event-related potentials indicating mental effort, attention, and emotion processing and -regulation before and after interventions.

##### Questionnaires

On the last day participants are asked to complete questionnaires at home to assess symptoms via e-mail. Here, the IDS-SR, Perseverative Thinking Questionnaire (PTQ) [66], Responses on Positive Affect Scale (RPA) [31], Emotion Regulation Questionnaire (ERQ) [67], Five Facet Mindfulness Questionnaire (FFMQ) [68], Leuven Adaptation of the Rumination on Sadness Scale (LARSS) [69], and the Positive and Negative Affect Schedule (PANAS) [70] are administered. After peri-intervention measurement weeks (T2, T4), we furthermore evaluate the interventions by asking participants how they have experienced the professional training and home exercises. Questionnaire data is collected and stored using REDCap.

The measurements include four sessions of two hours in the lab and 50 min of completing questionnaires at the end of the measurement week and around 60 min per day of completing the ESM questions and performing the mobile version of the SART for four weeks in total.

## Outcome measures

### Study aim 1

Primary outcomes for the first study aim in which we will examine changes in the content and dynamics of PC after interventions are within-subject changes, comparing pre- and peri-intervention measurements, in 1) self-reported measures of the content and dynamics of PC measured with ESM and the SART (using the following variables: rumination, occurrence of off-task thinking, valence of thoughts, temporal orientation of thoughts, self-relatedness of thoughts, and stickiness of thoughts), 2) HR and HRV during PC (i.e., negative, repetitive thinking) measured with ECG during ESM self-reports, and 3) EEG characteristics during PC measured in the context of the SART.

Secondary outcome measures that will be used to explore the role of sleep on the effectiveness of interventions in reducing the vulnerability for depressive relapse are 1) within-subject changes in objective and subjective sleep (measured as total sleep time, sleep quality, sleep onset latency) using actigraphy and ESM items about sleep and 2) within-subject changes in PC following the interventions. Moreover, we will examine changes in (residual) depressive symptoms and other depression-related measurements before and after the interventions (for questionnaires see Procedure) using within-subject comparisons.

### Study aim 2

To test whether changes in PC induced by fantasizing and mindfulness are different in rMDD versus ND participants, primary outcome measures are between-group differences in individual changes between pre- and peri-intervention measurements for both interventions (fantasizing and mindfulness) measured with 1) self-reported PC measures (ESM and SART), 2) HR and HRV during self-reported PC (ESM), and 3) EEG characteristics during PC measured with the SART.

### Study aim 3

Baseline individual characteristics of 100 participants (both ND and rMDD) for examining the magnitude of changes in PC following interventions include: 1) self-reported measures of PC measured with ESM and the SART, 2) HR and HRV measured with ECG during self-reported PC measured with ESM, 3) EEG characteristics during PC measured with the SART, and 4) resting state EEG. Predictors include mindfulness, rumination, emotion regulation strategies, psychiatric history, personality, dysfunctional attitudes, and childhood trauma.

## Statistical analyses

### Study aim 1

To test whether psychological and psychophysiological indices of PC are differentially affected by fantasizing vs. mindfulness, we will examine within-subject changes in pre- and peri-intervention measurements in the dynamics and content of self-reported PC, HR and HRV during self-reported PC, and electrophysiological correlates of PC and compare those between the fantasizing and mindfulness condition. We will check whether the relationships are linear or nonlinear. If the relationships are linear, we will perform linear mixed effect models (LME). If the relationships are non-linear, we will perform generalized additive models (GAMs) [71].

To examine changes in the dynamics and content of self-reported PC after interventions, several variables of PC over time will be examined. Specifically, we will consider rumination, occurrence of off-task thinking, valence of thoughts, temporal orientation of thoughts, self-relatedness of thoughts, and stickiness of thoughts. If interventions differently affect these variables of PC, we expect to find an interaction effect between intervention (fantasizing or mindfulness), session (pre- or peri-intervention), and time (time of ESM beep). For example, we hypothesized that mindfulness increases the frequency of present thoughts (i.e., temporal orientation of thought) in contrast to fantasizing where we do not expect this. If this would be the case, an interaction effect between intervention, session, and time on the temporal orientation of thoughts would be found. After running the LMEs or GAMs, visualization of the data will be used to further study the dynamics of the different changes over time.

To examine within-subject changes in pre- and peri-intervention measurements on the physiological correlates of PC, we will examine changes in HR and HRV and EEG voltage during self-reported PC. If interventions differently affect these physiological correlates of PC, we expect to find an interaction effect between intervention (fantasizing or mindfulness), session (pre- or peri-intervention), and time (in minutes to milliseconds).

To address the secondary objective about the role of sleep on the effectiveness of interventions in reducing PC, we will be using LMEs or GAMs depending whether the data are nonlinear. If sleep plays a role in whether interventions reduce PC, we expect to find a main effect of sleep (i.e., total sleep time, sleep quality and sleep onset latency) on changes in self-reported PC after the interventions.

To study the effects of the interventions on depressive symptoms using self-report questionnaires, a repeated measures ANOVA will be used with questionnaire scores (IDS-SR, PTQ, RPA, ERQ, FFMQ, LARSS, PANAS) administered as dependent variable and intervention (fantasizing or mindfulness) and session (pre- or peri-intervention) as independent variables. If interventions differentially affect depressive symptoms, we expect to find an interaction effect between intervention and session.

### Study aim 2

To study whether changes in PC by fantasizing and mindfulness are different in individuals with rMDD vs. ND controls, we will run the same analyses as for study aim 1, but with the variable group (rMDD or ND) added as an independent variable. Specifically, to examine the differential effects of self-reported PC, HR(V), and EEG, we will include session and intervention in interaction with group as independent variables in the model. If changes in PC after interventions are different in rMDD vs. ND individuals, we expect to find an interaction effect between group, session, and intervention on self-reported PC and its (neuro)physiological correlates measured with HR(V) and EEG.

### Study aim 3

For the third objective of exploring the role of individual characteristics on the effectiveness of the interventions at reducing PC, we will use baseline individual characteristics of participants assessed by means of a set of questionnaires (e.g., mindfulness, rumination, emotion regulation strategies, psychiatric history, personality, dysfunctional attitudes, childhood trauma etc.) and correlate those with changes in 1) self-reported measures of PC measured with ESM and the SART, 2) HR and HRV during reported PC, and 3) EEG characteristics during PC measured with the SART after mindfulness and positive fantasizing interventions. When significant correlations are found, we will do post-hoc moderation analyses to see how specific individual characteristics moderate changes in PC.

For all analyses, we will use a critical *p*-value of *p* < 0.05 that will be corrected for multiple comparisons using the Benjamini–Hochberg procedure [72]. Analyses will be performed using the statistical software R*.* LMEs and GAMs allow not only fixed effects, but also random effects. Random effects such as individual variability will therefore be added as well. In case non-significant effect are found for the LME analyses, Bayes Factors will be computed to find out whether this non-significant effect can be seen as proof for the H0 hypotheses (null effects) or whether this is due to the lack of reliability in the collected data [73]. Calculating Bayes Factors is not possible for GAMs, so this will only be conducted for LME models.

### Funding and ethics

The MINDCOG study is funded by the University of Groningen and the Dutch Research Council (NWO/NWA Idea generator grant 1228.191.473). The study design is in accordance with the Declaration of Helsinki (2013) and has been approved by the medical ethical committee of the University Medical Center Groningen (2019/537). Participants provide informed consent before participating in the screening session.

## Discussion

PC is an important factor in the vulnerability for depressive relapse [6, 7]. Several therapies have been shown effective in reducing depressive (residual) symptoms and the vulnerability for depressive relapse [23, 25, 26, 42, 74]. The mechanisms of change behind these therapies are however largely unknown. Usually, therapy efficacy is studied using questionnaires. How therapies exactly obtain their effects is not often studied. To study the mechanisms underlying the effects of relapse prevention treatments, detailed measurements are needed. In the current study, we combine both subjective and objective measurements for understanding how the therapeutic techniques of mindfulness and positive fantasizing change characteristics of PC and study whether specific techniques work better for individuals with specific characteristics. This can give insight in how mechanisms underlying these techniques interplay and obtain their effectiveness in different individuals. This allows us to learn more about depressive vulnerability and could potentially inform personalization of relapse prevention interventions for individuals in the future.

### Strengths

Our study is the first cross-over trial that includes several measurements at baseline and during intervention performance to track the underlying mechanisms of change of PC by therapeutic techniques, derived from existing therapies, in the vulnerability for depression. In a cross-over study design, participants are their own control, reducing the variability between groups and allowing for within-subject comparisons. By combining measurements in participants’ daily life (ESM), questionnaires, cognitive task, and physiological measurements at baseline and during intervention performance, we can get a detailed overview of how PC is affected by interventions on a behavioural, physiological, and cognitive level at different timescales. Several researchers have addressed the importance of within-subject analyses in psychiatry combing subjective and objective measurements [75, 76]. Moreover, the importance of studying the mechanisms of effects of existing psychological treatments has been addressed by several researchers [77]. Another strength of the study is the sample selection. We select our sample based on recent depressive episodes, current medication use, and previous experiences with mindfulness and/or positive fantasizing. Therefore, we study a sample that is highly vulnerable for depressive relapse in which confounding variables such as medication use and previous experiences are excluded. Furthermore, we are including baseline questionnaires capturing individual characteristics that might predict treatment effectiveness, allowing us to not only study the effects of the interventions but also what works best for whom.

### Challenges

At the moment of submitting this paper, we are in the middle of the data collection and had to deal with difficulties in the data collection due to the COVID-19 pandemic. The pandemic may have an effect on participants’ mood and PC [78]. In total, 47 participants were included during the COVID-19 pandemic. This may affect the generalizability of the results to the population.

Another feasibility challenge is that the study consists of many measurements and therefore we ask for quite some effort from the participant to complete the study, this can make it more difficult to find participants willing to participate in the research. Based on previous experience from the research team, the feasibility of the proposed study is predicted high (e.g., [37, 79, 80]).

## Conclusion

We described a multimodal cross-over study to examine the differential effects of a mindfulness versus positive fantasizing intervention technique on PC in individuals at risk for depressive relapse vs. ND individuals. In this study we will also examine what intervention techniques works best for whom by examining this on a mechanistic level. We aim to gain insight in the underlying mechanisms of change of these techniques on PC. Such insights may provide better understanding of how techniques are effective in reducing PC in individuals at risk for depressive relapse and how this differs from ND individuals. This could lead to better understanding in what makes some individuals vulnerable for depression and better-tailored personalized intervention strategies in the future.

### Data management and monitoring

All data received from participants will be processed in a strictly confidential fashion. Researchers other than those immediately involved in data-collection only have access to fully pseudo-anonymized files, which cannot be retraced to a specific individual. Data used for publication are also completely anonymous. All handling of personal data will comply with the European General Data Protection Regulation. The study will be monitored according to a monitoring plan including milestones guiding the research progress. Monitoring will be done by senior researchers involved in the project with lots of experience completing complex clinical studies using the similar measurements as also used in our study. Risk for the current study is estimated low, and monitoring will be performed in accordance.

### Supplementary Information

**Additional file 1:** **Supplementary Materials 1. **ESM items.

## Data Availability

No datasets were generated or analysed during the current study.

## Abbreviations

MDD: Major Depressive Disorder
PC: Perseverative cognition
ND: Never-depressed
PCT: Preventive cognitive therapy
HR: Heart rate
HRV: Heart rate variability
EEG: Electroencephalogram
rMDD: Remitted Major Depressive Disorder
ESM: Experience sampling method
ICG: Impedance cardiography
ECG: Electrocardiogram
DART: Dutch Adult Reading Test
DSM-5: Diagnostic Statistical Manual, version 5
IDS-SR30: Inventory of Depressive Symptomatology- Self-Report
SCID-5: Structured Clinical Interview for DSM-5
SART: Sustained Attention to Response Task
NEO-FFI: NEO Five-Factor Inventory
DAS-A: Dysfunctional Attitude Scale-form A
LEIDS-RR: Leiden Index of Depression Sensitivity-2nd revision
CTQ-SF: Childhood Trauma Questionnaire-Short Form
BVAQ: Bermond-Vorst Alexithymia Questionnaire
AES: Apathy Evaluation Scale
PTQ: Perseverative Thinking Questionnaire
RPA: Responses of Positive Affect Scale
ERQ: Emotion Regulation Questionnaire
FFMQ: Five Facet Mindfulness Questionnaire
LARSS: Leuven Adaptation of the Rumination on Sadness Scale
PANAS: Positive and Negative Affect Schedule
LME: Linear mixed effect models
GAMs: Generalized additive models
