# Supplementary material for: Understanding mechanisms of depression prevention: study protocol of a randomized cross-over trial to investigate mechanisms of mindfulness and positive fantasizing as intervention techniques for reducing perseverative cognition in remitted depressed individuals
Source: BMC Psychiatry. 2024 Feb 19;24:141. doi: 10.1186/s12888-024-05592-8 (PMC10877783; doi:10.1186/s12888-024-05592-8)
Supplement: Supplementary file 1 — Additional file 1: Supplementary Materials 1. ESM items. [file 12888_2024_5592_MOESM1_ESM.docx]

**Supplementary Materials 1 - ESM items**

ESM items are administered in Dutch but were translate for the purpose of the protocol paper.

1. Is this your first time filling in this questionnaire today? *[Original: Is dit de eerste keer vandaag dat u deze vragenlijst invult?]*

Ja -> if yes: continue

Nee -> if no: go to question 8

2. How was the quality of my sleep? *[Original: Hoe was de kwaliteit van mijn slaap?]*

VAS scale 1 -100

3. What time did I go to bed? *[Original: Hoe laat ging ik naar bed?]*hh:mm

4. Wat time did I try to fall asleep? *[Original: Hoe laat probeerde ik in slaap te vallen?]*hh:mm

5. How long did it take to fall asleep? *[Original: Hoe lang duurde het voordat ik in slaap viel?]*

… minutes

6. What time did I finally wake up? *[Original: Hoe laat werd ik uiteindelijk wakker?]*

hh:mm

7. I felt rested on waking up *[Original: Ik voelde me uitgerust bij het wakker worden]*

VAS scale 1 -100

8. At this moment, I feel cheerful *[Original: Op dit moment voel ik me opgewekt]*

VAS scale 1 -100

9. At this moment, I feel gloomy *[Original: Op dit moment voel ik me somber]*

VAS scale 1 -100

10. At this moment, I feel satisfied *[Original: Op dit moment voel ik me tevreden]*

VAS scale 1 -100

11. At this moment, I feel irritated *[Original: Op dit moment voel ik me geïrriteerd]*

VAS scale 1 -100

12. At this moment, I feel energetic *[Original: Op dit moment voel ik me energiek]*

VAS scale 1 -100

13. At this moment, I feel restless *[Original: Op dit moment voel ik me onrustig]*

VAS scale 1 -100

14. At this moment, I feel stressed *[Original: Op dit moment voel ik me gestrest]*

VAS scale 1 -100

15. At this moment, I feel anxious *[Original: Op dit moment voel ik me angstig]*

VAS scale 1 -100

16. At this moment, I feel listless *[Original: Op dit moment voel ik me lusteloos]*

VAS scale 1 -100

17. At this moment, I am thinking of: *[Original: Op dit moment denk ik aan]*

- The activity I am working on *[Original: De activiteit waar ik mee bezig ben]*

- Stimuli from the environment *[Original: Prikkels uit de omgeving]*

- How I feel at the moment *[Original: Hoe ik me op dit moment voel]*

- My personal concerns *[Original: Mijn persoonlijke zorgen]*

- I am daydreaming *[Original: Ik ben aan het dagdromen]*

- Other *[Original: Anders]*

18. At this moment, I am ruminating *[Original: Op dit moment ben ik aan het piekeren]*
VAS scale 1 -100

19. At this moment, my thought are not letting me go *[Original: Op dit moment laten mijn gedachten me niet los]*

VAS scale 1 -100

20. At this moment, I am not confortable with the thoughts I am experiencing [*Original: Op dit moment voel ik mij prettig bij de gedachten die ik ervaar]*

VAS scale 1 -100

21. At this moment, my thought are on *[Original: Op dit moment gaan mijn gedachten over]*

- The past *[Original: Het verleden]*

- The present *[Original: Het heden]*

- The future *[Original: De toekomst]*

22. At this moment, my thoughts *are [Original: Op dit moment zijn mijn gedachten]*

- Negative *[Original: Negatief]*

- Neutral *[Original: Neutraal]*

- Positive *[Original: Positief]*

23. At this moment, my thoughts are on *[Original: Op dit moment gaan mijn gedachten over]*

- Myself *[Original: Mijzelf]*

- Another *[Original: Een ander]*

- Neither *[Original: Geen van beiden]*

24. At this moment, I am easily distracted *[Original: Op dit moment ben ik snel afgeleid]*

VAS scale 1 -100

25. I feel like the rest of the day *[Original: Ik heb zin in de rest van de dag]*

VAS scale 1 -100

26. At this moment, I am *[Original: Op dit moment ben ik]*

- Alone *[Original: Alleen]* -> go to question 28

- In company *[Original: In gezelschap]* -> go to question 27

27. I find the company pleasant *[Original: Ik vind het gezelschap aangenaam]*

VAS scale 1 -100

28. I like being alone now *[Original: Ik vind het fijn om nu alleen te zijn]*

VAS scale 1 -100

29. Think of the most enjoyable event or activity since the last measurement moment. How enjoyable was this? *[Original: Denk aan de meest plezierige gebeurtenis of activiteit sinds het vorige meetmoment. Hoe plezierig was dit?]*

VAS scale 1 -100

30. How intens was this event *[Original: Hoe heftig was deze gebeurtenis?]*

VAS scale 1 -100

31. This of the most unpleasant event or activity since the last measurement moment. How unpleasant was this? *[Original: Denk aan de meest onplezierige gebeurtenis of activiteit sinds het vorige meetmoment. Hoe onplezierig was dit?]*

VAS scale 1 -100

32. How intense was this event? *[Original: Hoe heftig was deze gebeurtenis?]*

VAS scale 1 -100

33. Note any comments here *[Original: Noteer hier eventuele opmerkingen]*

Open field
